# Supplementary material for: The last resort antibiotic daptomycin exhibits two independent antibacterial mechanisms of action
Source: Nat Commun. 2025 Nov 24;16:10320. doi: 10.1038/s41467-025-65287-w (PMC12645038; doi:10.1038/s41467-025-65287-w)
Supplement: Supplementary file 1 — Supplementary Information [file 41467_2025_65287_MOESM1_ESM.pdf]

SUPPLEMENTARY INFORMATION

The last resort antibiotic daptomycin exhibits two independent antibacterial mechanisms of action

Authors: Jessica A. Buttress, Ann-Britt Schäfer, Alan Koh, Jessica Wheatley, Katarzyna Mickiewicz, Michaela Wenzel, and Henrik Strahl

Supplementary Table 1 Minimum inhibitory concentrations (MICs) of daptomycin against *B. subtilis* and *S. aureus* strains

Supplementary Table 2 Sample sizes and p-values

Supplementary Figure 1 Effects of daptomycin on cell growth in *S. aureus* and *B. subtilis*

Supplementary Figure 2 Effects of daptomycin on SYTOX Green fluorescence in *S. aureus*

Supplementary Figure 3 Inhibition of L-form growth by daptomycin

Supplementary Figure 4 *B. subtilis* AK092 L-forms still synthesise lipid II, whilst AK0197 L-forms do not

Supplementary Figure 5 Overview images of Nile red-stained untreated *B. subtilis*

Supplementary Figure 6 Overview images of Nile red-stained daptomycin-lipid clusters

Supplementary Figure 7 Deletion of *des* or *mprF* does not affect formation of fluid daptomycin-lipid clusters

Supplementary Table 1: Minimum inhibitory concentrations (MICs) of daptomycin against *B. subtilis* and *S. aureus* strains

Strain	Relevant genotype	MIC (µg/ml)
B. subtilis 168	WT	1.6
B. subtilis KS19	Δ*lytABCDEF*	1.6
B. subtilis CD11	Δ*liaH*	0.8
B. subtilis KS50	*des::kan*	1.6
B. subtilis AK0118	*mprF::kan*	0.8
S. aureus SH1000	WT	0.5

Gene and species names in italics.

Supplementary Table 2: Sample sizes and p-values

figure	panel	sample	sample size (n)
1	d	untreated	141
1	d	5 µg/ml, 5 min	124
1	d	5 µg/ml, 25 min	146
1	d	10 µg/ml, 5 min	147
1	d	10 µg/ml, 25 min	116
1	d	10 µM nisin, 5 min	124
2	b	untreated	125
2	b	5 µg/ml, 5 min	101
2	b	5 µg/ml, 25 min	144
2	b	10 µg/ml, 5 min	103
2	b	10 µg/ml, 25 min	127
2	b	10 µM, nisin, 5 min	128
3	b	10 min, untreated	309
3	b	10 min, 5 µg/ml daptomycin	281
3	b	10 min, 10 µg/ml daptomycin	249
3	b	10 min, 10 µM nisin	299
3	b	30 min, untreated	476
3	b	30 min, 5 µg/ml daptomycin	231
3	b	30 min, 10 µg/ml daptomycin	254
3	b	30 min, 10 µM nisin	288
3	b	60 min, untreated	706
3	b	60 min, 5 µg/ml daptomycin	449
3	b	60 min, 10 µg/ml daptomycin	378
3	b	60 min, 10 µM nisin	266
4	b	untreated (+ lipid II)	25
4	b	daptomycin 2 µg/ml (+ lipid II)	25
4	b	daptomycin 4 µg/ml (+ lipid II)	25
4	b	untreated (- lipid II)	25
4	b	daptomycin 2 µg/ml (- lipid II)	25
4	b	daptomycin 4 µg/ml (- lipid II)	25
4	d	untreated	78
4	d	daptomycin	67
4	d	gramicidin	65
5	b	untreated	142
5	b	1 µg/ml	73
5	b	2 µg/ml	73
5	b	4 µg/ml	85
5	b	8 µg/ml	56
5	b	gramicidin	40
5	d	untreated	147
5	d	1 µg/ml	171
5	d	2 µg/ml	176
5	d	4 µg/ml	106
5	d	8 µg/ml	112
5	d	gramicidin	105

figure	panel	one-way, unpaired ANOVA-comparison	p-value
3	b	DiSC3(5), 10 min, untreated vs 10 µg/ml daptomycin	0.85
3	b	DiSC3(5), 10 min, untreated vs 10 µM nisin	<0.0001
3	b	DiSC3(5), 30 min, untreated vs 10 µg/ml daptomycin	<0.0001
3	b	DiSC3(5), 30 min, untreated vs 10 µM nisin	<0.0001
3	b	DiSC3(5), 60 min, untreated vs 10 µg/ml daptomycin	<0.0001
3	b	DiSC3(5), 60 min, untreated vs 10 µM nisin	<0.0001
3	b	SYTOX, 10 min, untreated vs 10 µg/ml daptomycin	0.99
3	b	SYTOX, 10 min, untreated vs 10 µM nisin	<0.0001
3	b	SYTOX, 30 min, untreated vs 10 µg/ml daptomycin	0.50
3	b	SYTOX, 30 min, untreated vs 10 µM nisin	<0.0001
3	b	SYTOX, 60 min, untreated vs 10 µg/ml daptomycin	<0.0001
3	b	SYTOX, 60 min, untreated vs 10 µM nisin	<0.0001
4	b	untreated (+lipid II) vs daptomycin 2 µg/ml (+lipid II)	0.44
4	b	untreated (+lipid II) vs daptomycin 4 µg/ml (+lipid II)	<0.0001
4	b	untreated (+lipid II) vs untreated (-lipid II)	0.99
4	b	untreated (-lipid II) vs daptomycin 2 µg/ml (-lipid II)	0.99
4	b	untreated (-lipid II) vs daptomycin 4 µg/ml (-lipid II)	0.91
4	d	untreated vs daptomycin	<0.0001
4	d	untreated vs gramicidin	<0.0001
5	b	DiSC3(5), untreated vs 1 µg/ml	<0.0001
5	b	DiSC3(5), untreated vs 2 µg/ml	<0.0001
5	d	DiSC3(5), untreated vs 4 µg/ml	>0.99
5	d	DiSC3(5), untreated vs 8 µg/ml	<0.0001

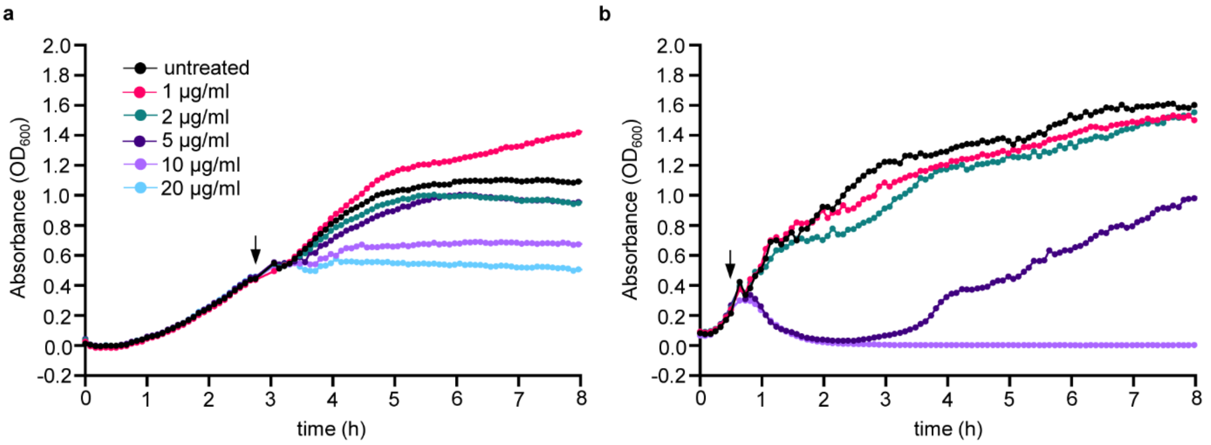

Supplementary Figure 1: Effects of daptomycin on cell growth in (a) *S. aureus* and (b) *B. subtilis*. Growth curves of cells in LB medium supplemented with 1.25 mM $CaCl_2$ and exposed to different daptomycin concentrations at OD_{600} of 0.3-0.4 (arrow). Strains used: *S. aureus* SH1000 and *B. subtilis* 168 (wild-type). The graphs depict representative data of two biological replicates.

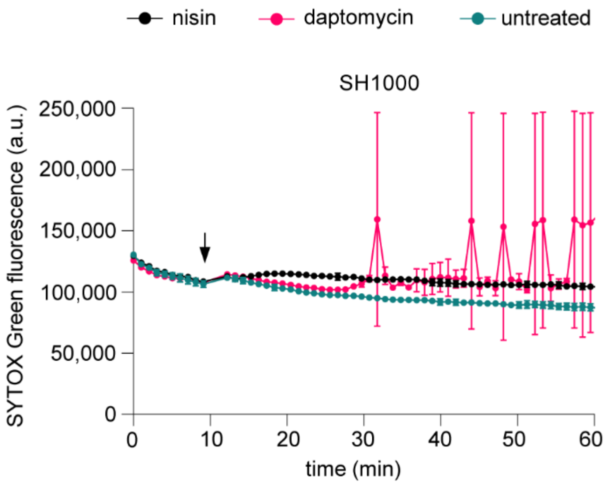

Supplementary Figure 2: Effects of daptomycin on SYTOX Green fluorescence in *S. aureus*. The effect of 10 µg/ml daptomycin on the fluorescence intensity of 1 µM SYTOX Green in *S. aureus* cell suspension at an initial OD_{600} of 0.5. The pore-forming lantibiotic nisin (10 µM) was used for positive control. The time point of antibiotic addition is indicated by an arrow. Data are represented as mean±SD of three technical replicates. Strain used: *S. aureus* SH1000. The graph depicts representative data of two biological replicates.

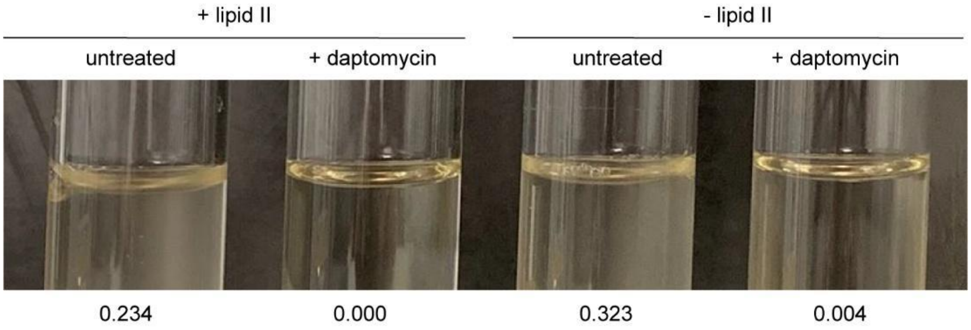

Supplementary Figure 3: Inhibition of L-form growth by daptomycin. *B. subtilis* L-forms able and unable to produce lipid II were untreated or treated with 10 µg/ml daptomycin and incubated at 30 °C for 72 h, until visible growth was observed. Figures at the bottom of the panel represent OD_{600} values. Strains used: *B. subtilis* AK092 (L-forms able to produce lipid II), *B. subtilis* AK0197 (L-forms unable to produce lipid II).

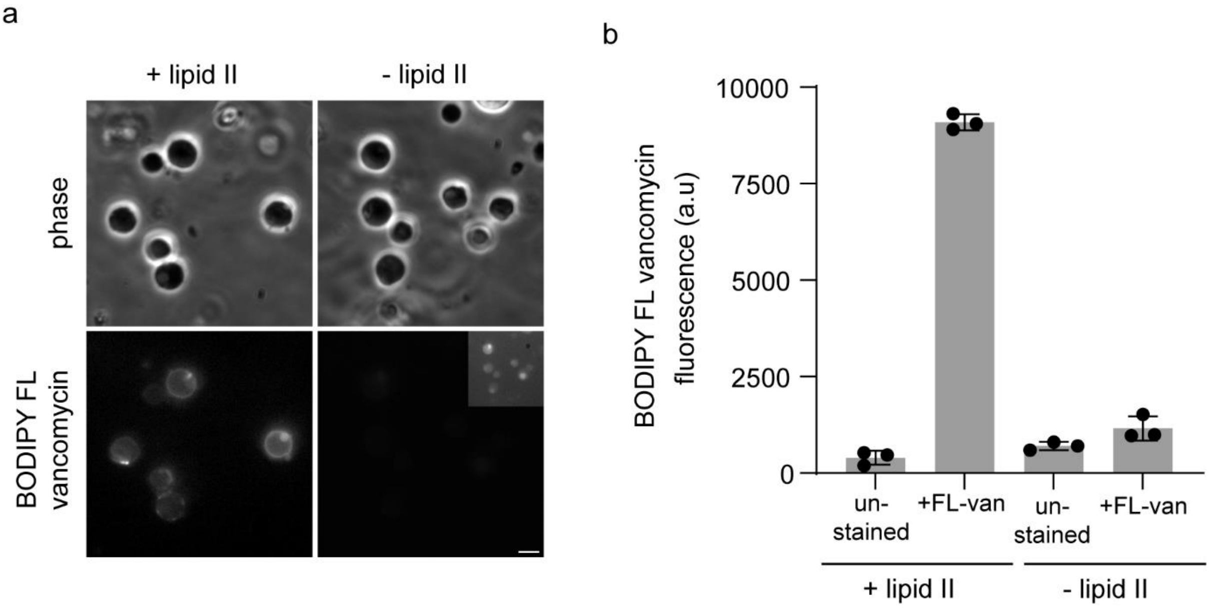

Supplementary Figure 4: *B. subtilis* AK092 L-forms still synthesise lipid II, whilst AK0197 L-forms do not. (a) Lipid II is not detectable in the Δ*uppS* L-form mutant (- lipid II) when stained with fluorescent vancomycin (BODIPY-FL vancomycin). Phase contrast (top panel) and BODIPY-FL vancomycin (bottom panel). The large images preserve the fluorescence intensity differences between the individual conditions. The small insert images serve to visualise the presence of cells in otherwise dark image fields. Scale bar, 3µm. (b) Quantification of overall BODIPY-FL vancomycin fluorescence in a plate reader with error bars indicating the standard deviation of at least three technical replicates from one experimental set. Strains used: *B. subtilis* AK092 (L-forms able to produce lipid II), *B. subtilis* AK0197 (L-forms unable to produce lipid II). The images and graphs depict representative data of three biological replicates.

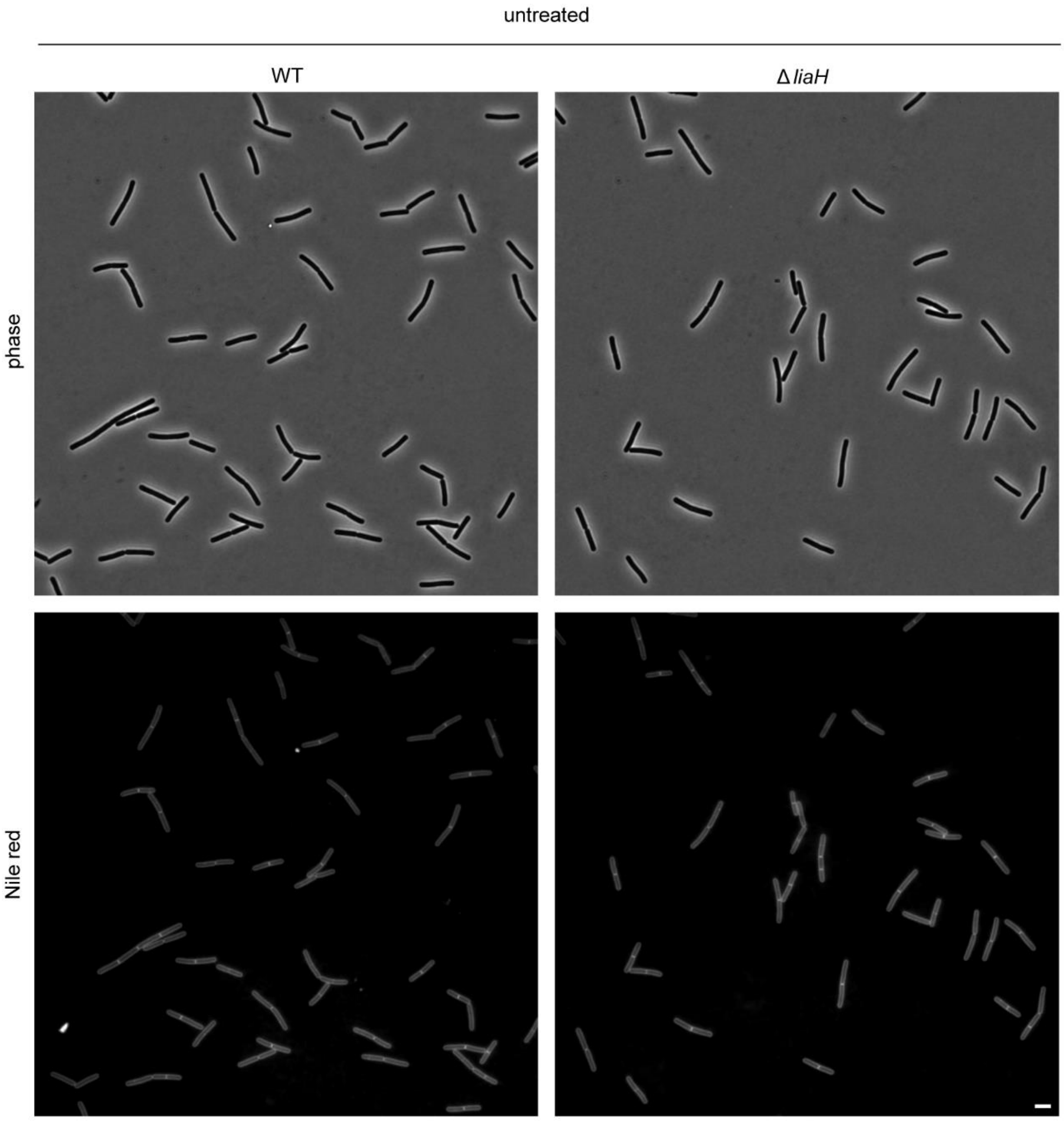

Supplementary Figure 5: Overview images of Nile red-stained untreated *B. subtilis*. Phase contrast and fluorescence microscopy of *B. subtilis* wild-type (WT) and Δ*liaH* cells stained with 1 µg/ml Nile red. Scale bar, 3µm. Strains used: *B. subtilis* 168 (wild-type) and CD11 (Δ*liaH*). The images depict representative data of three biological replicates.

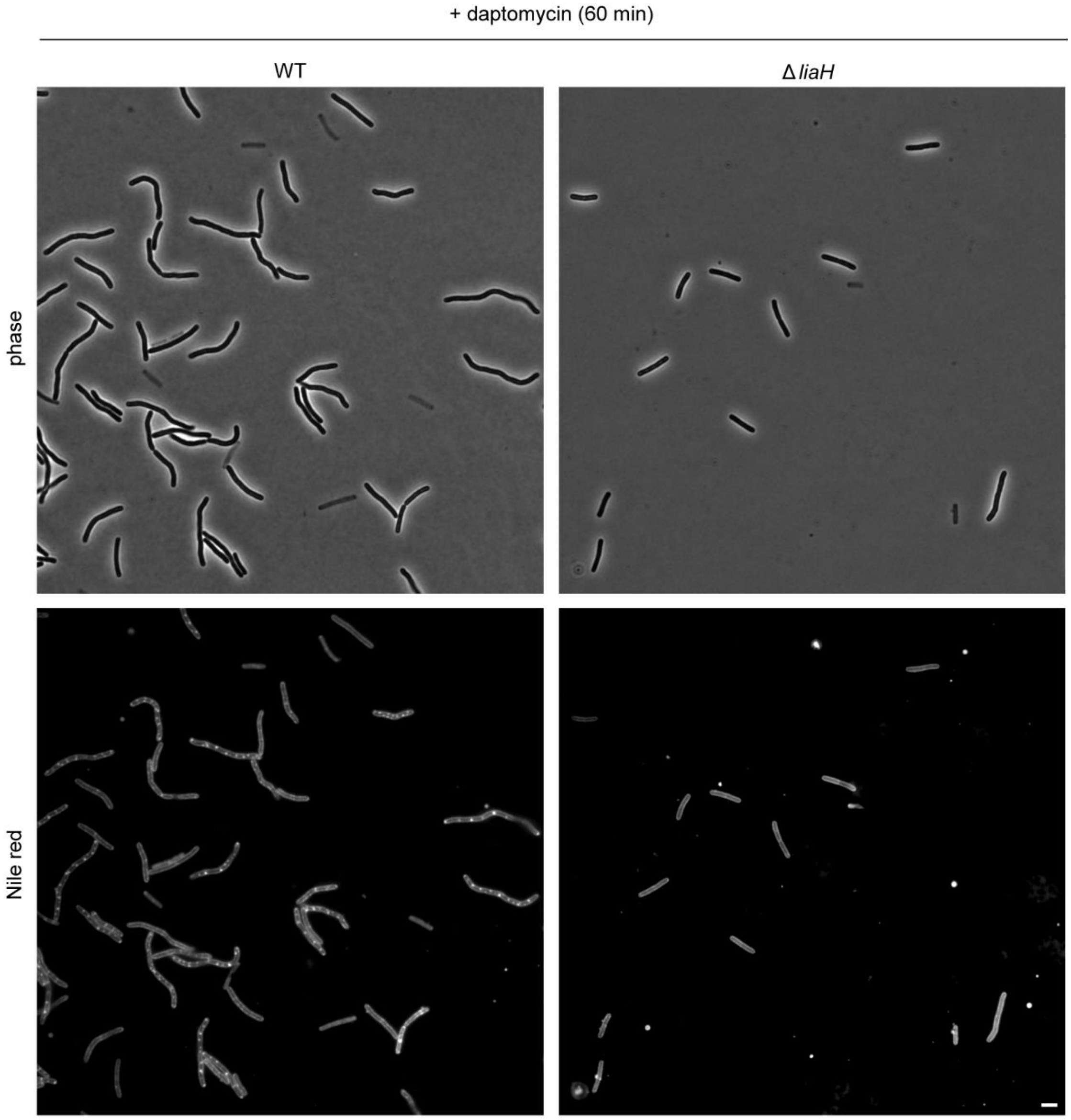

Supplementary Figure 6: Overview images of Nile red-stained daptomycin-lipid clusters. Phase contrast and fluorescence microscopy of *B. subtilis* wild-type (WT) and Δ*liaH* cells stained with 1 µg/ml Nile red and treated with 4 µg/ml daptomycin for 60 min. Scale bar, 3µm. Strains used: *B. subtilis* 168 (wild-type) and CD11 (Δ*liaH*). The images depict representative data of three biological replicates.

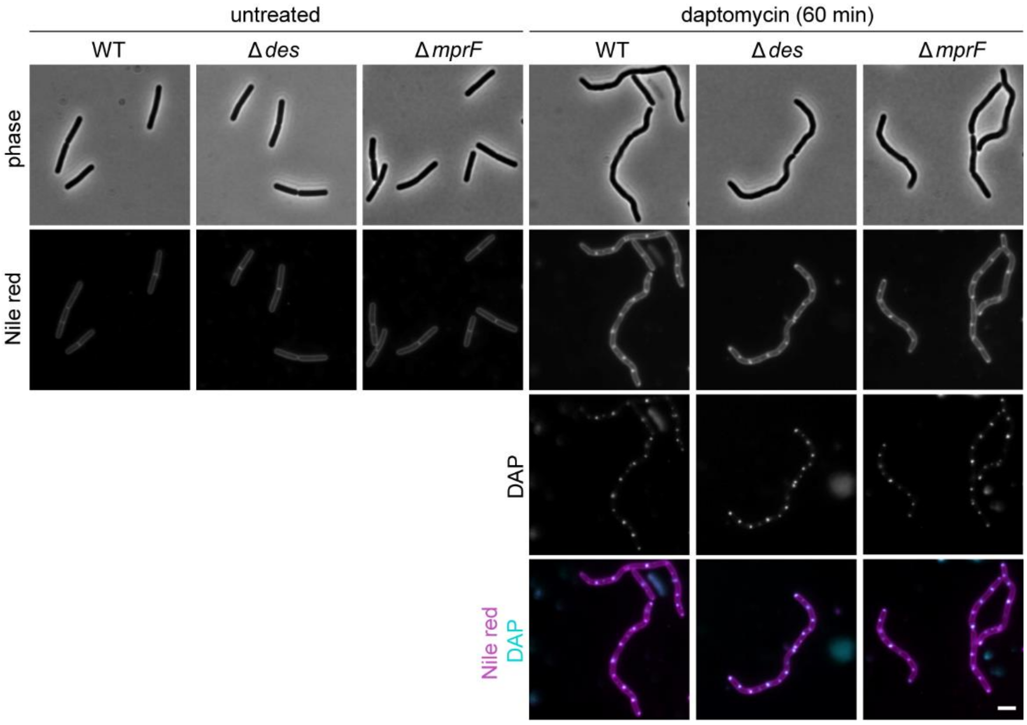

Supplementary Figure 7: Deletion of *des* or *mprF* does not affect formation of fluid daptomycin-lipid clusters. Phase contrast and fluorescence microscopy of *B. subtilis* wild-type (WT), Δ*des* and Δ*mprF* cells stained with 1 µg/ml Nile red and treated with 2 µg/ml daptomycin for 60 min. Please note that these experiments were carried out at reduced concentrations to limit the more extensive lysis observed for Δ*mprF*. Scale bar, 3µm. Strains used: *B. subtilis* 168 (wild-type), KS50 (Δ*des*) and AK0118 (Δ*mprF*). The images depict representative data of two biological replicates.
